# Transplant Trial Watch

**DOI:** 10.3389/ti.2022.10580

**Published:** 2022-06-01

**Authors:** Simon R. Knight

**Affiliations:** ^1^ Oxford Transplant Centre, Churchill Hospital, Oxford, United Kingdom; ^2^ Centre for Evidence in Transplantation, Nuffield Department of Surgical Sciences, University of Oxford, Oxford, United Kingdom

**Keywords:** heart transplantation, perfusion, attitudes, HCV, donor

To keep the transplantation community informed about recently published level 1 evidence in organ transplantation ESOT and the Centre for Evidence in Transplantation have developed the Transplant Trial Watch. The Transplant Trial Watch is a monthly overview of 10 new randomised controlled trials (RCTs) and systematic reviews. This page of Transplant International offers commentaries on methodological issues and clinical implications on two articles of particular interest from the CET Transplant Trial Watch monthly selection. For all high quality evidence in solid organ transplantation, visit the Transplant Library: www.transplantlibrary.com.

**Randomised Controlled Trial 1**

Clinician and Patient Attitudes Toward Use of Organs From Hepatitis C Viremic Donors and Their Impact on Acceptance: A Contemporary Review.

*by Fleetwood V. A., et al. Clinical Transplantation. 2021; 35 (12):e14519.*

## Aims

This study aimed to evaluate the attitudes of clinicians and patients regarding the use of organs from hepatitis C viremic donors and their impact on acceptance.

## Interventions

A literature search was conducted on PubMed, MEDLINE, and SCOPUS. Studies were selected for inclusion by two independent reviewers. Data were extracted by the primary author.

## Participants

8 studies were included in the review.

## Outcomes

The outcomes of interest included knowledge of HCV-specific outcomes, HCV-specific concerns, willingness to accept viremic organs and factors that contributed to acceptance or non-acceptance.

## Follow-up

Not applicable.

## CET Conclusion

This is an interesting review concerning patient attitudes towards receiving organs from Hepatitis C positive donors. Multiple databases were searched, and papers were assessed in duplicate, although one author conducted the data extraction. Eight articles were included (6 survey questionnaires, 1 semi-structured interview and 1 conjoint analysis). The paper provides a narrative review of the included articles, summarised in key themes. The authors have done well to summarise a difficult topic and provide a synthesis of the included studies. There is however no assessment of the quality of the included papers.

## Funding Source

No funding was received for this study.

**Randomised Controlled Trial 2**

Long-Term Outcomes After Heart Transplantation Using *Ex Vivo* Allograft Perfusion in Standard Risk Donors: A Single-Center Experience.

*by Chen Q., et al. Clinical Transplantation 2022; e14591.*

## Aims

This study aimed to assess the long-term outcomes of heart transplant patients that received allografts preserved using the Organ Care System (OCS) versus standard cold storage (CS).

## Interventions

Participants were randomised to receive allografts preserved with either CS or OCS.

## Participants

38 heart transplant candidates.

## Outcomes

The primary outcomes were 8-year overall survival and freedom from cardiac allograft-related death up to 8 years. Secondary outcomes were 8-year freedom from cardiac allograft vasculopathy (CAV), freedom from non-fatal major adverse cardiac events and freedom from rejections.

## Follow-up

8 years.

## CET Conclusions

This paper reports the long-term outcome from hearts randomised in the randomised PROCEED II study at a single centre. Previous publications of the PROCEED II study have already shown non-inferior short-term outcomes comparing perfusion on the OCS device to cold storage on ice. The study as a whole included 130 patients randomised in a 1:1 fashion, and this single-centre follow up reports on only 38. As such, this latest report is underpowered to identify all but the most obvious of clinical differences and the authors acknowledge this limitation. Follow-up in this cohort of 38 was acceptable, at 92%, which equates to 3 lost-to follow up. Recipients in the cold-storage arm were significantly older, by 8 years. There was no significant difference in overall survival at median follow up of 8.4 years and no difference in cardiac allograft vasculopathy. The study outcomes should be viewed in the context of a highly selected donor and recipient population, with any potential benefits more likely to show themselves when using extended criteria donors.

## Trial Registration

ClinicalTrials.gov—NCT00855712.

## Funding Source

Non-industry funding.

## Clinical Impact Summary

Utilisation of deceased donor cardiothoracic organs is typically lower than those of abdominal organs (1). This has led to interest in methods for *ex-vivo* preservation and viability assessment, which have the potential to prolong preservation times, recondition organs and improve outcomes by allowing assessment prior to transplantation.

The first randomised controlled trial of normothermic *ex-vivo* cardiac preservation (PROCEED II) was reported in the Lancet in 2015 (2). The study randomised 130 transplant recipients to receive a heart either stored using conventional static cold storage (SCS) or preserved using the Organ Care System (OCS) perfusion device. The authors reported non-inferiority of perfused hearts, with no measurable difference in patient or graft survival despite longer overall preservation times in the OCS group. Of note, 5 hearts were discarded due to preservation parameters in the OCS group, but despite the potential advantages of discarding suboptimal organs, there was no measured clinical benefit (3). All hearts in the study had to be suitable for either arm and were relatively low-risk, meaning that any impact on organ utilisation cannot be assessed.

In a recent paper published in Clinical Transplantation, Chen et al. report long-term outcomes in 38 patients from a single participating centre from the trial (4). Eight-year survival was numerically lower in the OCS group (57.9% vs. 73.7%, *p* = 0.24) but not meeting statistical significance in this small sample. The apparent excess mortality in the OCS group seemed mainly related to events that are difficult to attribute to the preservation method (e.g., CMV infection or malignancy), supported by a lack of difference in the rate of graft-related mortality (84.2% in both groups).

In contrast to the survival data, there was numerically higher freedom from coronary allograft vasculopathy (CAV; 89.5% vs. 67.8%) and non-fatal major cardiac events (89.5% vs. 67.5%) in the OCS group. Differences in CAV rate may relate to the shorter cold-ischaemic times in the OCS group, reducing ischaemia reperfusion injury.

Overall, the small sample size means that firm conclusions are difficult to draw and this study is unlikely to have a significant impact on clinical practice. It would perhaps have been more useful to compile long-term outcomes from all patients in the original study to increase statistical power and see if the trends seen here were borne out in other centre’s data.

Use of the OCS device is feasible and likely safe, but there is limited evidence of clinical benefit in standard-risk hearts. Whether *ex-vivo* perfusion will have a greater utility in preservation and viability assessment of hearts from more marginal donors remains to be seen.
